# Lysyl Oxidases Expression and Breast Cancer Progression: A Bioinformatic Analysis

**DOI:** 10.3389/fphar.2022.883998

**Published:** 2022-06-21

**Authors:** Sofia Ramos, Sandra Ferreira, Ana S. Fernandes, Nuno Saraiva

**Affiliations:** CBIOS, Universidade Lusófona’s Research Center for Biosciences and Health Technologies, Lisbon, Portugal

**Keywords:** lysyl oxidase, breast cancer, pharmacological inhibitors, gene expression, bioinformatics, immune infiltration

## Abstract

LOX (Lysyl oxidase) and LOX like 1–4 (LOXL1–4) are amine oxidases that catalyse the cross-linking of elastin and collagen in the extracellular matrix (ECM). This activity can facilitate cell migration and the formation of metastases. Consequently, inhibition of these enzymes and, in particular of LOXL2, has been suggested as a therapeutic strategy to prevent breast cancer metastasis. Although medicinal chemistry studies have struggled to specifically inhibit LOXL2, the importance of selectivity in this context is not clear. To explore the role of each LOX in breast cancer and consequently their potential as biomarkers or therapeutic targets, a bioinformatic-based approach was followed. The expression profile of *LOX*s, the putative associations among mRNA expression from each *LOX* and clinical observations, the correlation between expression of *LOX* enzymes and other genes, and the association between expression of *LOX*s and the tumour infiltrates were assessed for breast cancer. Overall, the patient outcome and the characteristics of breast tumours with *LOX*, *LOXL1* and *LOXL2* upregulation is distinct from those with high expression of *LOXL3* and *LOXL4*. Additionally, the expression correlation between *LOX*s and other genes involved in cellular processes relevant for cancer biology, also reveals a similar trend for *LOX*, *LOXL1* and *LOX2*. This work further supports the relevance of LOXL2 as a breast cancer progression biomarker and therapeutic target. We speculate that while the impact of LOXL3 inhibition may vary with breast cancer subtype, the therapeutical inhibition of LOX, LOXL1 and LOXL2 but not of LOXL4 may be the most beneficial.

## Introduction

Breast cancer is the most common type of cancer in women, with approximately 2.6 million cases diagnosed annually (Sung et al., 2021). Current therapeutic approaches to treat advanced breast cancer with distant organ metastases are not considered effective, resulting in very low patient survival rates. This strongly contrasts with early-stage non-metastatic disease where the available therapies are able to cure ∼70–80% of patients (Harbeck et al., 2019). Presently, treatment decisions take into account the high level of molecular heterogeneity of these tumours, consequently increasing patient life expectancy. In this context, the molecular heterogeneity of breast cancers should be taken into account when developing new therapeutic approaches (Testa et al., 2020).

The human Lysyl oxidase (LOX), and lysyl oxidase like-1 to 4 (LOXL1–LOXL4) belong to the lysyl oxidase family. The primary function of these enzymes is to catalyse the cross-linking of elastin and collagen in the extracellular matrix (ECM) (Rucker et al., 1998). Altered expression of genes from this family influences the remodelling of ECM components and consequently tissue stiffness. Therefore, these proteins can impact cancer cell proliferation, survival, invasion, migration, epithelial to mesenchymal transition, ultimately influencing tumour progression (Vallet et al., 2021). This is supported by data linking expression dysregulation of LOX enzymes with metastasis and tumour survival rates in some cancers (Ahn et al., 2013; Cox et al., 2015; Salvador et al., 2017; Yu et al., 2020; Ferreira et al., 2021). Consequently, LOX and LOXL1–4 enzymes have been suggested as potential druggable targets to prevent breast cancer metastasis (Cox et al., 2016; Ferreira et al., 2021). Various compounds have been developed with different inhibitory activities against LOXs (Ferreira et al., 2021). Depending on their structure, the inhibitors developed so far can be specific for LOXL2, dual inhibitors for LOX/LOXL2, dual inhibitors for LOXL2/LOXL3, or pan-LOX inhibitors (Ferreira et al., 2021). However, the importance of selectively inhibiting each of these enzymes in the various breast cancer subtypes is not clear.

Here we use The Cancer Genome Atlas (TCGA) Breast Invasive Carcinoma (BRCA) data (https://portal.gdc.cancer.gov/projects/TCGA-BRCA) to explore the relation between the expression of each *LOX* gene at the mRNA level with breast cancer patient survival, expression of genes involved in cellular mechanisms associated with cancer progression and tumour infiltrates in several breast cancer subtypes. Thus, providing a basis to clarify the potential usefulness of each LOX as progression biomarker or therapeutic target in breast cancer.

## Methods

To explore the relevance of each *LOX* gene in BRCA and its subtypes [Basal-like, human epidermal growth factor receptor 2+ (HER2+), Luminal A (LumA) and Luminal B (LumB)], a bioinformatic approach was followed using the TCGA BRCA data set for mRNA expression. The expression profile of *LOX* family enzymes in BRCA and its subtypes and the association of this expression with patient survival were obtained using GEPIA2–Gene Expression Profiling Interactive Analysis (http://gepia2.cancer-pku.cn/#analysis) (Tang et al., 2019). Differential expression between normal and tumour tissues was determined by a one-way ANOVA test. To explore the association between *LOXs* individual expression levels and the prognosis of BRCA patients, Kaplan-Meier survival analysis with Log-rank test, and Cox Proportional Hazard (PH) Model were generated using the median cut-offs of tumour *LOX*s mRNA levels for both overall survival (OS) and disease-free survival (DFS). Cox proportional hazard ratios (HR) from low and high expressions were plotted in a heatmap. Differences between Kaplan-Meier curves were analysed by the Log rank test.

To study the relation between the expression of *LOX*s and other genes, the one hundred genes with the highest Pearson correlation for each *LOX* were obtained from the TCGA BRCA data set using both GEPIA2 and UALCAN, a web-portal to perform in-depth analyses of TCGA gene expression data (Chandrashekar et al., 2017) (http://ualcan.path.uab.edu). Non-protein coding mRNAs were excluded from this analysis and only genes commonly found in both platforms were included. To compare the correlation of each selected gene with the remaining *LOX*s, Pearson´s correlation values were obtained from GEPIA2. Genecard and Uniprot were used to categorize each selected gene based on their functional characterization. Genes were clustered into four groups: 1) cell migration, adhesion, and ECM regulation; 2) cell survival and proliferation; 3) angiogenesis and tumour proliferation; 4) others (other functions).

The association between expression of *LOX*s and abundance of immune infiltrates in BRCA and its subtypes was evaluated using the TCGA data-based platform TIMER2.0 (http://timer.cistrome.org/) (Li et al., 2020). Spearman’s correlations were calculated based on the algorithm EPIC (Estimating the Proportion of Immune and Cancer cells). This bioinformatic tool predicts the fraction of different cell types from bulk tumour gene expression data, by integrating gene expression profiles from each major non-malignant cell type and renormalizing based on cell-type-specific mRNA content (Racle et al., 2017). The Spearman correlations coefficients obtained were plotted in a heatmap.

## Results and Discussion

### Lysyl Oxidase Family Gene Expression and Breast Cancer Patient Survival

**FIGURE 1:**
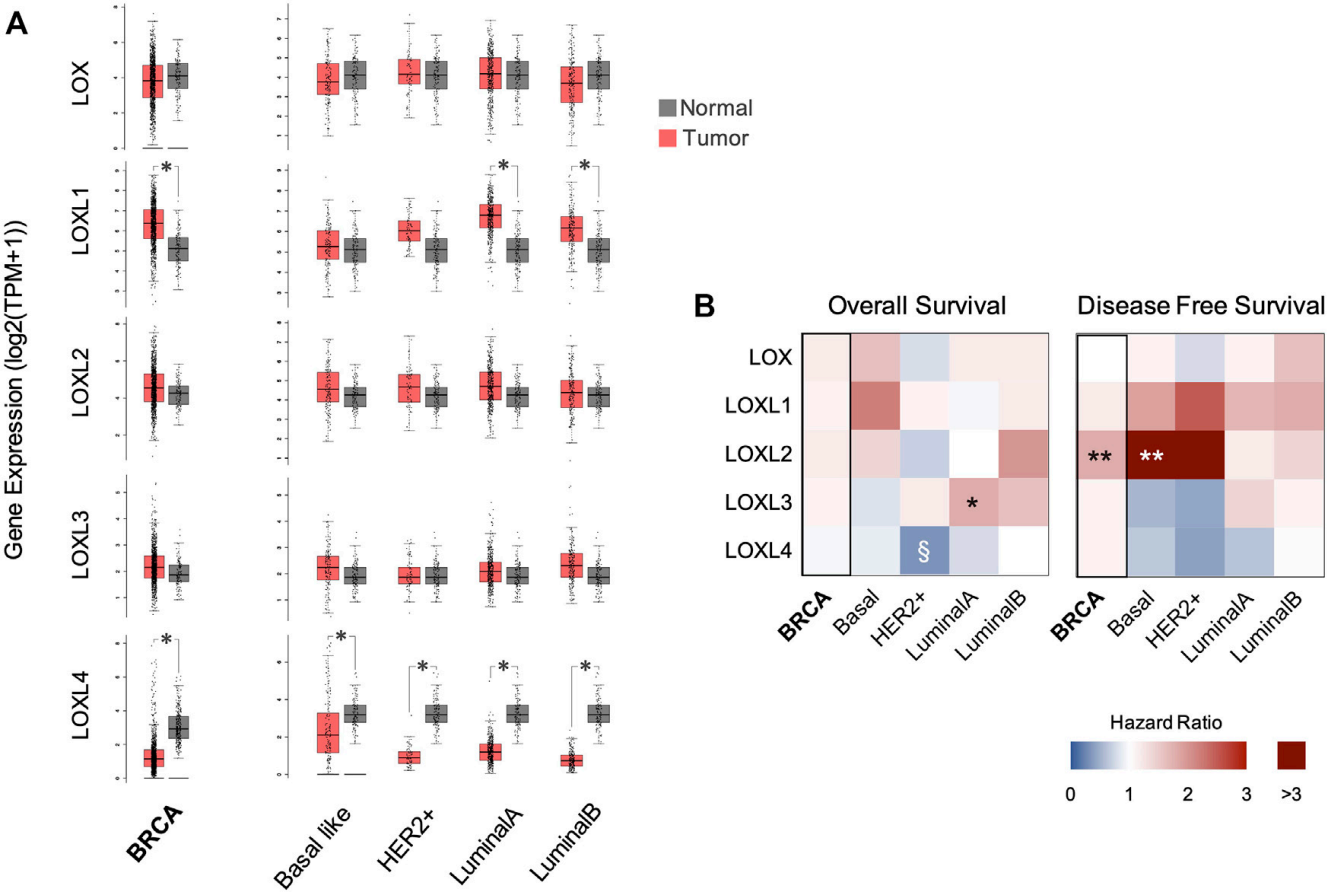
*LOX* family gene expression and breast cancer patient survival. **(A)** *LOX*s mRNA expression profiling comparative analysis between BRCA (*n* = 1085) or its subtypes (Basal-like *n* = 135; HER2+ *n* = 66; LumA *n* = 415; and LumB *n* = 194) and normal (*n* = 112) tissue samples. *p < 0.01 (one-way ANOVA). **(B)** Overall survival and Disease-free survival Hazard ratio in patients with high tumour levels of *LOX*s mRNA calculated from Kaplain-Meier curves using the Cox PH Model. Hazard ratios using *LOX*s expression median were calculated for BRCA and its subtypes. *p < 0.05, **p < 0.01, ^§^ *p* = 0.051 (Logrank test).

To characterize the expression levels of *LOX* and *LOXL1-4* on normal and breast cancer tissues, the *LOX*s mRNA levels from TCGA BRCA data-set were analysed. While *LOX* expression is not significantly altered, higher average levels of *LOXL1*, *LOXL2*, and *LOXL3* were found in breast cancer tissues when compared with normal tissues (Figure 1A). This is particularly evident in the LumA and LumB subtypes. Despite this, only *LOXL1* showed significant differences between expression in normal and tumour tissues in BRCA and LumA and LumB subtypes. Other studies have reported an upregulation of *LOXL2* in invasive/metastatic breast cancer cells when compared with poorly invasive/nonmetastatic breast cancer cells (Kirschmann et al., 2002). Thus, highlighting the important role of this protein in breast cancer progression (Salvador et al., 2017). Clinical and preclinical data also suggest that higher LOXL2 expression is associated with invasiveness of Basal-like breast cancer cells (Ahn et al., 2013). Unlike *LOXL1-3*, the levels of *LOXL4* mRNA were significantly lower in breast cancer and in all analysed subtypes, when compared with normal tissue (Figure 1A).

Considering the role of LOX enzymes in cellular events related with cancer progression, the associations between *LOX*s mRNA levels and patient survival were assessed. In breast cancers, the correlations found were more pronounced in DFS than in OS (Figure 1B). While LOXL2 and to a minor extent LOXL1 increased expression was generally correlated with a poorer outcome as measured by an increased hazard ratio (HR) for DFS, the upregulation of LOXL4 was associated with lower HR values (Figure 1B). Effectively, our results concur with Ahn et al. (2013) that showed that dysregulation of LOXL2 expression in Basal-like breast cancer contributes to a poor prognosis and to appearance of distant metastasis.

Patients with low tumour mRNA levels of *LOXL4* showed a reduction in OS and DFS HR, particularly in the HER2+ subtype (Figure 1B). LOXL4 expression dysregulation can have a progressive or repressive impact depending on the cancer type, the context and the tumour stage (Wong et al., 2011; Shao et al., 2019). Several studies show that LOXL4 downregulation is mostly associated with cellular events related with tumour progression and with enhanced tumour growth and metastasis in different cancer models (Wu et al., 2007; Shao et al., 2019) and specifically in breast cancer (Choi et al., 2017; Yin et al., 2020). Despite the unclear role of LOXL4 in tumour biology (Choi et al., 2017), a weak LOXL4 expression can lead to the remodelling of the ECM, induction of collagen synthesis, deposition, and to structural changes. These modifications can promote tumour growth and metastasis and are associated with poor clinical outcomes in triple-negative breast cancer (Choi et al., 2017).

### Correlation Between Lysyl Oxidases Expression and Other Genes in Breast Cancer

**FIGURE 2:**
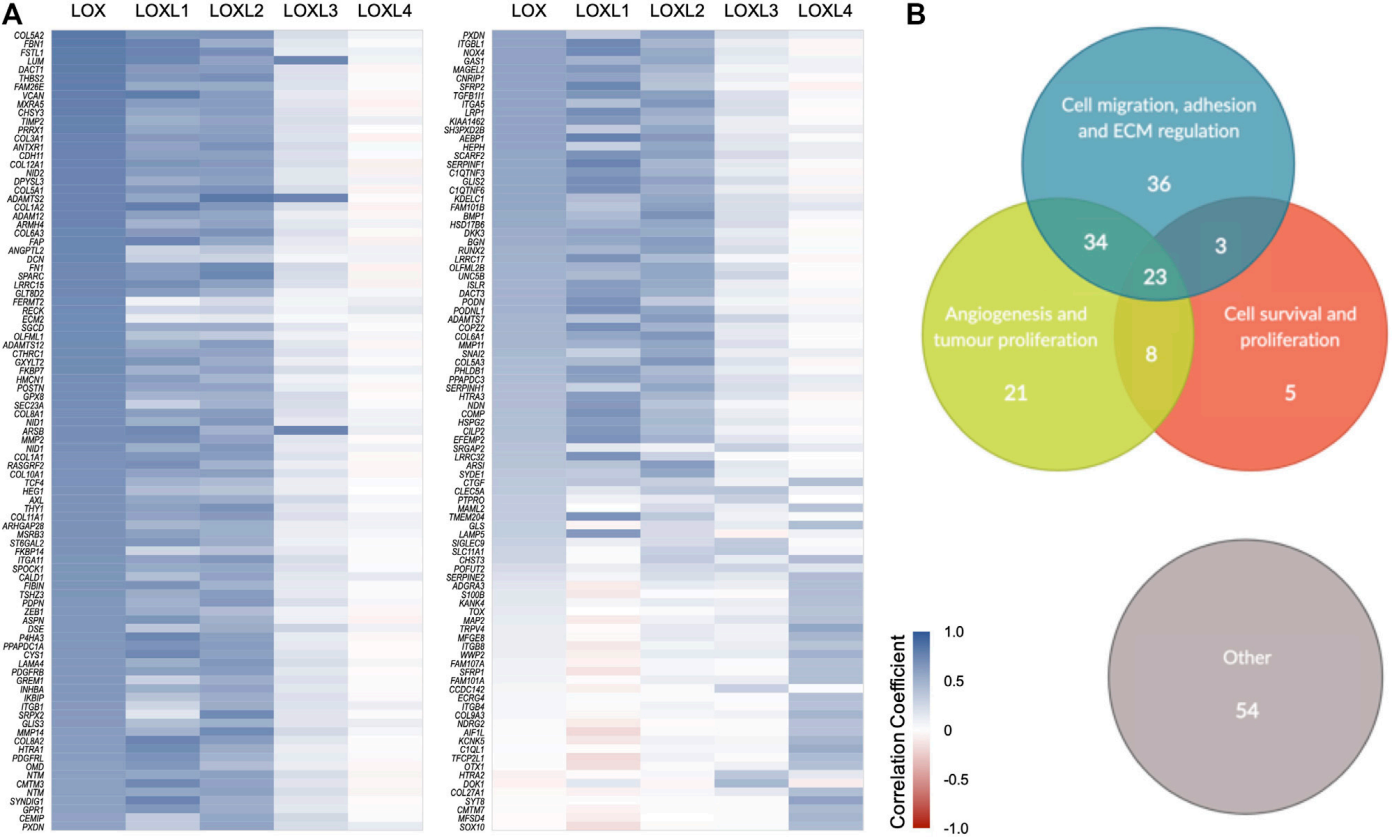
Correlation between the expression of *LOX*s and other genes in breast cancer. **(A)** Heat map showing Pearson’s correlation coefficients between mRNA expression of *LOX* family genes and other genes. For each *LOX*, the one hundred most strongly correlated genes in breast cancer were collected from GEPIA and UALCAN, and only those common to both platforms were selected. Blue gradient represents a positive Pearson’s correlation and red gradient a negative correlation. **(B)** Genes were clustered according to their described functions, highlighting specific groups that are relevant for breast cancer progression-related events.

To explore the relation between the altered expression of *LOX*s and the mRNA levels of other genes in breast cancer, the genes more strongly correlated with *LOX*s expression in breast cancer were listed and their correlation coefficients plotted (Figure 2A). The genes gathered from this analysis were clustered according to their described functions. Interestingly, the great majority of the genes found have assigned biological functions related with tumour progression (Figure 2B). Considering the role of LOXs in ECM remodelling, it is not surprising that altered expression of several of these genes is associated with profound changes in the expression of genes related to cell migration, adhesion, ECM regulation and angiogenesis (Figure 2). The expression interdependency of some genes identified using our strategy have been previously demonstrated in breast cancer cell lines where *LOX*s were knocked down. Saatci O, et al. 2020 (Saatci et al., 2020) silenced *LOX* gene using siRNAs and observed a downregulation in fibronectin 1 (*FN1*) and integrin subunit alpha 5 (*ITGA5*) mRNA levels. In a different study, the knockdown of *LOX* lead to the reduction of Snail Family Transcriptional Repressor 2 (SNAI2) mRNA and protein expression levels (Boufraqech et al., 2016). The silencing of LOXL2 decreased the expression of cadherin 11 (CDH11) protein (Moreno-Bueno et al., 2011). These experimental observations are in agreement with the gene expression correlations found in the TCGA BRCA dataset.

The correlation trend observed between expression of *LOX*, *LOXL1* and *LOXL2* and the expression of the majority of genes analysed was often similar. Interestingly, *LOXL4* presented an opposite trend in most cases. Additionally, the expression of *LOXL3* appears not to strongly correlate with any group of genes in this context. These results are in line with the observed association between *LOX*s gene expression and patient survival.

### Association Between Tumour Lysyl Oxidases Gene Expression and Breast Tumour Infiltrates

**FIGURE 3:**
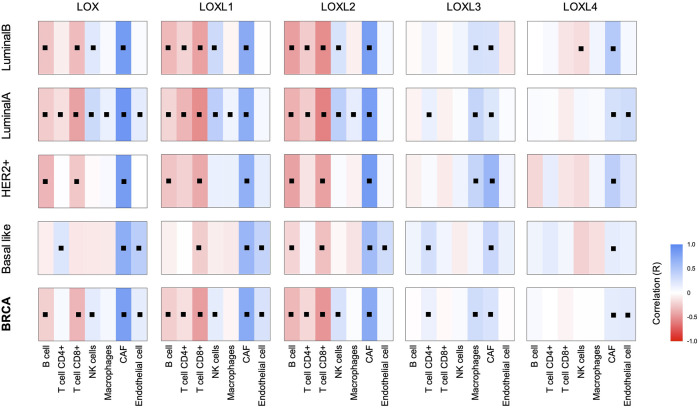
*LOX*s expression and breast cancer tumour infiltrates. The correlations between expression of *LOX* family members and the abundance of immune infiltrates in BRCA and its subtypes were calculated based on the EPIC (Estimating the Proportion of Immune and Cancer cells) algorithm. Blue gradient represents a positive Spearman’s correlation and red gradient a negative correlation, ■ *p* < 0.05 (Spearman). Number of samples in each group: BRCA = 1100; Basal-like = 191; Her2+ = 82; LumA = 568; LumB = 219.

Tumour microenvironment is a key aspect of cancer biology that strongly controls tumour progression. Considering the numerous roles of this family of proteins that are associated with ECM remodelling, we hypothesise that LOXs have a great impact on tumour infiltrates. Effectively, previous works have demonstrated that LOX secreted in the hypoxic tumour environment of invasive breast cancer can contribute to the recruitment of inflammatory cells to a distant site, promoting the formation of premetastatic niches (Cox et al., 2012). To explore the relationship of *LOX*s expression on breast cancer tumour microenvironment, the prevalence of various tumour-infiltrating cells was calculated using immune deconvolution and marker gene-based methods (Figure 3). Data obtained revealed an association between the increased expression of *LOX* enzymes and the presence of Cancer-associated fibroblasts (CAFs) in all breast cancer subtypes. This increase in CAFs was more pronounced in the case of *LOX*, *LOXL1* and *LOXL2* upregulation, when compared with *LOXL3* or *LOXL4.* Although with some exceptions, the expression of *LOX*, *LOXL1* and *LOXL2* was globally negatively associated with B, T CD4^+^ and T CD8^+^ cell infiltration. In breast cancer, less immunogenic tumours are typically associated with a poor prognosis (Dieci et al., 2021). This is in agreement with the worst outcome observed in Figure 1B for breast cancer patients with higher expression of *LOXL1* and *LOXL2* genes.

The relation between *LOXL3* and *LOXL4* expression and immune infiltrates is more modest, especially for B and T cells. Despite that, a positive correlation between LOXL4 expression and macrophage infiltration was found, as also observed by Yin et al. (2020). Globally, tumours with *LOX*, *LOXL1* and *LOX2* upregulation have a distinct tumour cell infiltrating profile from tumours with *LOXL3* or *LOXL4* increased gene expression.

Equally to *LOX*s gene expression and correlation with genes related to breast cancer progression, the patterns of tumour cell infiltration observed for *LOX*, *LOXL1* and *LOXL2* are comparable, and distinct from those of *LOXL3* and *LOXL4*.

## Future Implications

Many of the molecular mechanisms involved in breast cancer invasion and metastasis are still unclear. Considering the impact of breast cancer metastasis in patient outcome, increased therapeutic specificity that accounts for the molecular heterogeneity of the tumour is highly desirable. In that sense, LOX and LOXL1-4 are interesting candidates as novel targets to modulate breast cancer progression. Data presented here highlight the importance of Lysyl oxidases gene expression and its association with breast cancer patient survival and relapse. Specifically, the increased expression of *LOX*, *LOXL1* and *LOXL2* appears to be correlated with similar trends in terms of patient survival, tumour infiltrates and correlation with expression of genes involved in tumour-related processes. Contrarily to *LOXL3*, for which strong correlations were not found, or to *LOXL4* that was associated with opposite trends. Previous studies have demonstrated that, contrarily to LOXL2, LOXL4 overexpression has an inhibitory effect on cancer proliferation and progression-related events in different cancer models (Wu et al., 2007; Choi et al., 2017; Shao et al., 2019). Thus, the results obtained are in agreement with the limited available data describing the impact of LOXs in cancer.

Several authors have proposed the LOX family proteins, mostly LOXL2 as therapeutic targets in breast cancer treatment (Moreno-Bueno et al., 2011), in accordance with the data presented here. The current targeting strategy focuses on inhibiting the enzymatic activity of LOX proteins. The small molecule inhibitors developed so far present distinct selectivity towards different enzymes of the LOXs family. However, a rational basis to pursue a specific selectivity profile in the drug development process, tailored for each potential therapeutic use, is still missing. The work presented here contributes to fill this gap for the case of breast cancer. Overall, we speculate that while the impact of LOXL3 inhibition may vary with breast cancer subtype, the specific therapeutical inhibition of both LOXL1 and LOXL2 but not of LOXL4 may be beneficial in breast cancer. Therefore, these data provide a rational basis for the drug development process of novel LOXs inhibitors aimed for breast cancer treatment.

## Data Availability

Publicly available datasets were analyzed in this study. This data can be found here: https://www.cancer.gov/about-nci/organization/ccg/research/structural-genomics/tcga (TCGA, BRCA dataset).

## Abbreviations

LOX, lysyl oxidase; LOXL1-4, LOX like1-4; ECM, extracellular matrix; TCGA, the cancer genome atlas; BRCA, breast invasive carcinoma; GEPIA, gene expression profiling interactive analysis; HER2+, human epidermal growth factor receptor 2+; LumA, luminal A; LumB, luminal B; OS, overall survival; DFS, disease free survival; HR, hazard ratio; CAFs, cancer-associated fibroblasts.
